# Editorial: Rhythm in human cognition and action: Health and pathology

**DOI:** 10.3389/fpsyg.2022.1047825

**Published:** 2022-10-19

**Authors:** Charles-Etienne Benoit, Laura Ferreri, Carlotta Lega, Floris T. van Vugt

**Affiliations:** ^1^Inter-University Laboratory of Human Movement Biology, Univ Lyon, University Claude Bernard Lyon 1, Villeurbanne, France; ^2^Department of Brain and Behavioral Sciences, University of Pavia, Pavia, Italy; ^3^Laboratoire d'Etude des Mécanismes Cognitifs, Université Lumière Lyon 2, Lyon, France; ^4^Department of Psychology, University of Milano-Bicocca, Milan, Italy; ^5^Psychology Department, International Laboratory for Brain, Music, and Sound Research, University of Montreal, Montreal, QC, Canada; ^6^Haskins Laboratories, Yale University, New Haven, CT, United States

**Keywords:** rhythm, music, cognition, action, movement, cognitive therapy, social interaction, synchronization

As a group of young scientists building their careers doing research in the cognitive science of music, we saw over the past decade how the field grew more legitimate. In this line of research, the study of rhythm in humans presents a long and rich scientific history which increasingly demonstrates the positive impacts of rhythm-driven enhancement at both neural and behavioral levels.

When we first launched the special issue “*Rhythm in Human Cognition and Action: Health and Pathology*”, we wished to highlight the wide range of benefits that engaging with rhythmical events can generate: as a means to boost motors, cognitive, social and communication abilities. Looking back on the work in this special issue, we can clearly state that our goal was met: several research teams around the globe representing all these areas of research answered the call (51 authors published altogether 10 articles, representing 34 affiliations from 14 countries). Through this editorial article, we wish to survey the cohesion in which rhythm permeates so many aspects of our life. In particular, the current Research Topic collects several articles offering an updated view of the plurality of approaches in the study of rhythm in human cognition, ranging from experimental to clinical and social perspective. The issue will include a series of original reports and reviews that offer a comprehensive picture of the multifaceted interplay between rhythm and human functioning.

Indeed, perception, action, and cognition are strongly linked: the article by Fiveash et al. brings into light that rhythm perception involves strong auditory-motor connections that can be enhanced with movement. They investigated if a visual cue moving in time with regular rhythms could enhance the rhythmic priming effect. They demonstrate that on the contrary, visual over auditory cues could hinder the effect by potentially creating a dual-task situation.

The article published by Cochen De Cock et al. echoes these findings and shows that with the use of an auditory cue in the form of music can be used in a personalized rhythmical training to reinforce gait patterns in Parkinson's patients. This approach can also reduce fear of falling and improve the patients' overall quality of life. This was made possible by using a portable phone-based application aligning the musical tempo in real time to optimize gait.

The work by Ravi et al. further presents the link between rhythm and locomotion. They argue that movement circuitry contributes to the continuous regulation of our walking rhythm, even in the presence of perturbations that can destabilize locomotion. Temporal adaptation to perturbations provides an understanding of the link between rhythm and balance while their results extend on the sensorimotor synchronization paradigm toward improving our understanding of an individual's resilience to perturbations and potential fall risk.

The importance of sensorimotor synchronization in health is explored in more depth in the review article by von Schnehen et al. showing that such a therapeutic approach is able not only to benefit in a large array of neurocognitive disorders, but also to bring positive changes in healthy aging. This work also synthesizes the brain and cognitive mechanisms involved in these known benefits.

The article by Verga et al. expands on this neurocircuitry and demonstrates that traumatic brain injuries to these important brain areas create dysfunctional timing. They suggest that basic co-occurring perceptual and motor timing impairments may factor into a wide range of daily activities.

One such daily activity is our ability to move. The article by Ferreri et al. emphasizes that a regular rhythmic stimulation increases people's ability to anticipate future events in time and to move their body in space. Their findings support the idea that temporal predictions driven by a regular auditory stimulation are grounded in a perception-action system integrating temporal and spatial information.

The transferability of sensorimotor synchronization can be extended to the area of language. The article by Kertész and Honbolygo demonstrates that tapping to music predicts literacy skills of first-grade children. Their results show that phonological awareness, spelling and reading accuracy were associated with the musical tasks while reading fluency was predicted. Language by itself is a social endeavor: more facets of our social interactions are influenced by rhythm.

Indeed, moving together in time affects human social affiliation and cognition. The work by Basile et al. investigates if these effects extend to on-line video meetings through the lens of empathy or the theory of the mind. They concluded that participants in synchronous movement rated feeling greater closeness and similarity to their partners relative to an asynchronous condition, and that this influences social affiliation measures.

When looking at social interactions, people coordinate not only at the behavioral, but also at the physiological and neural levels, and that this coordination gives a temporal structure to the individual and social dynamics. Farrera and Ramos-Fernandez review the evidence for the existence of group-level rhythmic patterns that result from social interactions and argue that the complexity of group dynamics can lead to temporal regularities that cannot be predicted from the individual periodicity leading to an emergent collective rhythm.

Finally, from a mathematical modeling standpoint, it has been observed that the frequency of participants' oscillations reduces when compared to that acquired in solo. Calabrese et al. aim at capturing this phenomenon by proposing three alternative modifications of the standard Kuramoto model often employed to model human synchronization.

Taken together, these works show how rhythm broadly affects human functioning. This question is approached through a wide range of different perspectives, from experimental studies to modeling and reviews. We want to thank all the authors for their splendid contributions that push the boundaries in our understanding of the complex interactions between many facets of human experience.

## Author contributions

All authors listed have made a substantial, direct, and intellectual contribution to the work and approved it for publication.

## Funding

CEB was supported in part by the Reg. and Molly Buck award. LF is supported by ANR-20-CE28-0008. CL is supported by a Bial Foundation grant (241/2020).

## Conflict of interest

The authors declare that the research was conducted in the absence of any commercial or financial relationships that could be construed as a potential conflict of interest.

## Publisher's note

All claims expressed in this article are solely those of the authors and do not necessarily represent those of their affiliated organizations, or those of the publisher, the editors and the reviewers. Any product that may be evaluated in this article, or claim that may be made by its manufacturer, is not guaranteed or endorsed by the publisher.

